# Supplementary material for: Improving the Safety and Continuity Of Medicines management at Transitions of care (ISCOMAT): protocol for a process evaluation of a cluster randomised control trial
Source: BMJ Open. 2020 Nov 24;10(11):e040493. doi: 10.1136/bmjopen-2020-040493 (PMC7689064; doi:10.1136/bmjopen-2020-040493)
Supplement: Author's manuscript [file bmjopen-2020-040493.draft_revisions.pdf]

BMJ Open is committed to open peer review. As part of this commitment we make the peer review history of every article we publish publicly available.

When an article is published we post the peer reviewers' comments and the authors' responses online. We also post the versions of the paper that were used during peer review. These are the versions that the peer review comments apply to.

The versions of the paper that follow are the versions that were submitted during the peer review process. They are not the versions of record or the final published versions. They should not be cited or distributed as the published version of this manuscript.

BMJ Open is an open access journal and the full, final, typeset and author-corrected version of record of the manuscript is available on our site with no access controls, subscription charges or pay-per-view fees (http://bmjopen.bmj.com).

If you have any questions on BMJ Open's open peer review process please email
info.bmjopen@bmj.com

BMJ Open

Improving the Safety and Continuity Of Medicines management at Transitions of care (ISCOMAT): protocol for a process evaluation of a cluster randomised control trial

Journal:	*BMJ Open*
Manuscript ID	bmjopen-2020-040493
Article Type:	Protocol
Date Submitted by the Author:	14-May-2020
Complete List of Authors:	Powell, Catherine; University of Bradford Faculty of Life Sciences, School of Pharmacy and Medical Sciences<br>Breen, Liz; University of Bradford Faculty of Life Sciences, School of Pharmacy and Medical Sciences; Bradford Institute for Health Research, Yorkshire and Humber Patient Safety Translational Research Centre<br>Fylan, Beth; University of Bradford Faculty of Life Sciences, School of Pharmacy and Medical Sciences; Bradford Institute for Health Research, Yorkshire and Humber Patient Safety Translational Research Centre<br>Ismail, Hanif; University of Bradford Faculty of Life Sciences, School of Pharmacy and Medical Sciences<br>Alderson, Sarah; University of Leeds School of Medicine<br>Gale, Chris; University of Leeds, Leeds Institute of Cardiovascular and Metabolic Medicine<br>Gardner, Peter; University of Bradford Faculty of Life Sciences, School of Pharmacy and Life Sciences<br>Farrin, Amanda; University of Leeds, Clinical Trials Research Unit<br>Alldred, David ; University of Leeds, School of Healthcare; Bradford Institute for Health Research, Yorkshire and Humber Patient Safety Translational Research Centre
Keywords:	Heart failure < CARDIOLOGY, CARDIOLOGY, Clinical trials < THERAPEUTICS, STATISTICS & RESEARCH METHODS

SCHOLARONE™
Manuscripts

I, the Submitting Author has the right to grant and does grant on behalf of all authors of the Work (as defined in the below author licence), an exclusive licence and/or a non-exclusive licence for contributions from authors who are: i) UK Crown employees; ii) where BMJ has agreed a CC-BY licence shall apply, and/or iii) in accordance with the terms applicable for US Federal Government officers or employees acting as part of their official duties; on a worldwide, perpetual, irrevocable, royalty-free basis to BMJ Publishing Group Ltd ("BMJ") its licensees and where the relevant Journal is co-owned by BMJ to the co-owners of the Journal, to publish the Work in this journal and any other BMJ products and to exploit all rights, as set out in our licence.

The Submitting Author accepts and understands that any supply made under these terms is made by BMJ to the Submitting Author unless you are acting as an employee on behalf of your employer or a postgraduate student of an affiliated institution which is paying any applicable article publishing charge ("APC") for Open Access articles. Where the Submitting Author wishes to make the Work available on an Open Access basis (and intends to pay the relevant APC), the terms of reuse of such Open Access shall be governed by a Creative Commons licence – details of these licences and which Creative Commons licence will apply to this Work are set out in our licence referred to above.

Other than as permitted in any relevant BMJ Author's Self Archiving Policies, I confirm this Work has not been accepted for publication elsewhere, is not being considered for publication elsewhere and does not duplicate material already published. I confirm all authors consent to publication of this Work and authorise the granting of this licence.

For peer review only

Improving the Safety and Continuity Of Medicines management at Transitions of care (ISCOMAT): protocol for a process evaluation of a cluster randomised control trial

Corresponding author

Dr Catherine Powell

Room M24

Richmond Building

School of Pharmacy and Medical Sciences,

University of Bradford,

Richmond Rd, Bradford BD7 1DP, UK

c.powell2@bradford.ac.uk

01274 236338

Authors

Dr Catherine Powell, School of Pharmacy and Medical Sciences, University of Bradford, Richmond Road, Bradford, BD7 1DP, UK. https://orcid.org/0000-0001-7590-0247

Dr Liz Breen, School of Pharmacy and Medical Sciences, University of Bradford, Richmond Road, Bradford, BD7 1DP, UK. Yorkshire and Humber Patient Safety Translational Research Centre. Bradford Institute for Health Research, Temple Bank House, Bradford, BD9 6RJ Bradford Institute for Health Research, Bradford, UK. https://orcid.org/0000-0001-5204-1187

Dr Beth Fylan, School of Pharmacy and Medical Sciences, University of Bradford, Richmond Road, Bradford, BD7 1DP, UK. Yorkshire and Humber Patient Safety Translational Research Centre. Bradford Institute for Health Research, Temple Bank House, Bradford, BD9 6RJ, UK. https://orcid.org/0000-0003-0599-4537

Dr Hanif Ismail, School of Pharmacy and Medical Sciences, University of Bradford, Richmond Road, Bradford, BD7 1DP, UK. https://orcid.org/0000-0002-7885-6648

Dr Sarah Alderson, School of Medicine, Worsley Building, University of Leeds, Leeds LS2 9JT, UK. https://orcid.org/0000-0002-5418-0495

Professor Chris P Gale, Leeds Institute of Cardiovascular and Metabolic Medicine, University of Leeds, Leeds, UK. https://orcid.org/0000-0003-4732-382X

Professor Peter Gardner, School of Pharmacy and Medical Sciences, University of Bradford, Richmond Road, Bradford, BD7 1DP, UK. https://orcid.org/0000-0002-8799-0443

Professor Amanda J Farrin, Clinical Trials Research Unit, Leeds Institute of Clinical Trials Research, University of Leeds, Leeds, LS2 9JT, UK. http://orcid.org/ 0000-0002-2876-0584

Professor David P Alldred, University of Leeds, School of Healthcare, Baines Wing, Leeds LS2 9DA, UK. Yorkshire and Humber Patient Safety Translational Research Centre. Bradford Institute for

For peer review only

Health Research, Temple Bank House, Bradford, BD9 6RJ, UK. https://orcid.org/0000-0002-2525-4854

Word count (excluding title page, abstract, references, figures and tables): 3614

Key words: Heart failure, Cardiology, Clinical trials, Statistics and Research Methods

ABSTRACT

Introduction: A key priority for the UK National Health Service (NHS) and patients is to ensure that medicines are used safely and effectively. However, medication changes are not always optimally communicated and implemented when patients transfer from hospital into community settings. Heart failure is a common reason for admission to hospital. Patients with heart failure have a high burden of morbidity, mortality and complex pharmacotherapeutic regimes. The Improving the Safety and Continuity Of Medicines management at Transitions of care (ISCOMAT) programme comprises a cluster randomised controlled trial which will test the effectiveness of a complex behavioural intervention aimed at improving medications management at the interface between hospitals discharge and community care. We will conduct a rigorous process evaluation to inform interpretation of the trial findings, inform implementation of the intervention on a wider scale and aid dissemination of the intervention.

Methods and analysis: The process evaluation will be conducted in six purposively selected intervention sites (i.e. hospital trusts and associated community pharmacies) using a mixed-methods design. Fidelity and barriers/enablers of implementation of the Medicines at Transitions Intervention (MaTI) will be explored using observation, interviews (20 patients, 40 healthcare professionals), surveys and routine trial data collection on adherence to MaTI. A parallel mixed analysis will be applied. Qualitative data will be thematically analysed using Framework analysis and survey data will be analysed descriptively. Data will be synthesised, triangulated and mapped to the Consolidated Framework for Implementation Research (CFIR) where appropriate. The process evaluation commenced on June 2018 and is due to end on February 2021.

Ethics and dissemination: Approved by Research Ethics Committee and the UK Health Research Authority REC: 18/YH/0017 / IRAS: 231431. Findings will be disseminated via academic and policy conferences, peer-reviewed publications and social media.

Trial registration number: ISRCTN: 66212970/

ARTICLE SUMMARY

Strengths and limitations of this study

- An evaluation of a cluster randomised controlled trial of a complex behavioural intervention for a high priority healthcare issue.
- Views of patients and health professionals working across both primary and secondary care.
- A key strength is our mixed methods and the flexibility of our approach in light of emerging issues from the study and the shifts in healthcare practice and policy.
- The process evaluation is limited to six purposively selected intervention sites.

For peer review only

INTRODUCTION

Heart failure presents a major challenge to healthcare systems globally.[1] In the UK it is thought to affect the lives of over 920,000 people.[2] In a study of four million individuals, heart failure cases have been found to increase 12% from 2002 to 2014.[3] Myocardial infarction has been identified as a risk for heart failure.[4] Heart failure is treated by a combination of medication, lifestyle changes and interventional surgery depending on the severity of the condition. Typically, the pharmacological treatment pathway involves a combination of medicines that are titrated to the optimal level that patients can tolerate. If not managed well (or if the patient has not been diagnosed and treated in primary care) they may be admitted to hospital for treatment and to be stabilised. Approximately 5% of all emergency hospital admissions are for heart failure.[5] Ongoing treatment plans following discharge from hospital may not be fully implemented due to failures in communication between healthcare providers or a lack of specialist staff in the community.[6, 7] Patients may not understand their medicines, what they are for and why they need to take them because, for example, medical language may create a gap in patients' understanding.[8] Patients may therefore deteriorate, which can result in a re-admission to hospital or death.[9]

The ISCOMAT study

ISCOMAT is a five-year National Institute for Health Research (NIHR) funded research programme, which aims to optimise the way heart failure patients are supported with their medicines when they move from hospital to home. This may contribute to improving patients' health through helping them better understand and use their medicines. ISCOMAT also aims to improve the way health professionals work together in order to improve communication and optimise medicines use when patients return home. Similar international studies have examined patient-centred care transitions for patients hospitalized for heart failure and found clinical outcomes did not improve. ISCOMAT is designed for the UK health system and may show improvements in clinical outcomes in this setting.[10] In earlier work packages, we explored the resilience of the medicines management

pathway for heart failure and then used Experienced Based Co-Design to develop the Medicines at Transitions Intervention (MaTI) with patients and professionals.[8, 11] The MaTI was designed to make best use of medicines and reduce harm through: the provision of information to the patient; enhanced communication between hospital and the patient's community pharmacist; and increased engagement of the community pharmacist post discharge. We have provided limited information about the intervention here to avoid potential contamination of the ongoing trial. A more detailed description of the MaTI intervention will be published alongside feasibility testing. The cluster randomised controlled trial is testing the effectiveness of the MaTI and will recruit an average of 50 patients over approximately 12 months from cardiology ward(s) in 42 acute NHS trusts across England (target n=2100 patients). Sites are randomised to either treatment as usual or to the MaTI. A site co-ordinator is responsible for organising the trial and implementation of the MaTI at each site.

The process evaluation

Process evaluations are increasingly carried out alongside randomised controlled trials.[12, 13] They are particularly well suited to trials of complex interventions in multiple sites where the intervention may be implemented differently throughout sites and help us to understand more about the practical problems encountered and how they were resolved. We will follow the Medical Research Council (MRC) recommendations and guidance on process evaluations.[13] The process evaluation will examine the effect of organisational context and setting on intervention delivery, and how the intervention is best implemented. There are no predefined methods that a process evaluation must adopt although they typically involve mixed methods.[14, 15] Although the process and outcomes evaluation will be conducted by separate teams, the trial and process evaluation teams will meet regularly to have oversight.

Aims and objectives

The aims of the process evaluation are to inform interpretation of the trial findings, inform implementation of the intervention on a wider scale (for example, other long term conditions) and aid potential future implementation of the intervention.

Our objectives at each of the six process evaluation sites are to:

- Determine the degree to which the intervention is delivered (internal fidelity)
- Explore and explain the relationship between intervention implementation and the trial outcomes
- Identify barriers and facilitators for the successful implementation and roll out of the intervention (should the intervention be effective)

For peer review only

PROCESS EVALUATION METHODS

Study design

In addition to the aforementioned MRC guidance,[13] we will draw on other relevant literature,[16, 17] in particular the Consolidated Framework for Implementation Research (CFIR).[18] The CFIR is a conceptual framework that was developed to guide systematic assessment of multilevel implementation contexts to identify factors that might influence intervention implementation and effectiveness.[19] The MaTI intervention is implemented at multiple levels at each site including trusts, secondary and primary health care professionals and patients. The CFIR framework generates a knowledge base for implementation across multiple settings within five major domains: intervention characteristics, inner setting, outer setting, individual characteristics, and implementation process. Domains will be explored and additional relevant theoretical frameworks applied as appropriate. We will also draw on human resource management evaluation, specifically the ability, motivation and opportunity (AMO) model,[20, 21] and capability, opportunity, and motivation (COM-B) model.[22] These models are complementary and highlight how policy interventions that require changes in staff behaviour are shaped by their ability to work in different ways, in terms of skills, their level of motivation and their opportunities to change practice.

The study design is a parallel mixed synthesis study using quantitative and qualitative data from six intervention sites. Methods will involve non-participant observations, semi-structured interviews and surveys. In order to capture data on barriers and facilitators to implementation we will collect data from health professionals working along the patient pathway (from both secondary and primary care), patients and community pharmacists. We will also utilise fidelity data on adherence to MaTI that is being collected within each intervention site in the trial. We will explore and explain the relationship between intervention implementation and the trial outcomes in the six process evaluation sites through analysing secondary outcome data. These data will indicate whether the intervention improves patient understanding of their medicines and satisfaction with medicines-related care at two and six weeks post-discharge and twelve months post-registration from the Patient Experience Survey (PES) (a validated item from Coleman's transition measure [23]), alongside observations and implementation data collected as part of the process evaluation. Process evaluations need to be flexible in the context of the ongoing trials they are evaluating and we will record and describe adaptations to this protocol when the findings are published.

Sampling and recruitment

We aim to purposively sample six intervention sites based on the following criteria:

- University and non-university hospitals

For peer review only

- Method for transferring medicines discharge information to community pharmacists' e.g. electronic system, post, telephone
- Sites located across different geographic areas of England

Within each of the six selected sites we will recruit patients and staff for interviews and surveys. We will also conduct observations at each of these sites. The sampling approach will be iterative and following sampling of one-to-two pilot sites; consideration of the initial data in relation to fidelity will be made.

Patient interviews

Twenty patients in total will be sampled for interviews (three to four patients across each of the six sites). We will adopt a purposive sampling strategy to meet our target characteristics in terms of gender, age, length of diagnosis, and fidelity to intervention. After piloting, we may need to modify our approach to enable diversity in relation to our sampling criteria. Patients approached will be those that have participated in the trial and received the MaTI intervention.

Observations of intervention wards

We will seek permission to conduct non-participant observations of staff at ward level (two and a half hours per site). Staff members will be provided with information sheets and a script for informing patients of the researchers' presence. A maximum of two researchers will be present at any one time. Staff will have the opportunity to opt out of the observation. A potential limitation of conducting overt observation is the Hawthorne effect by which individuals being observed may alter their behaviour because they are aware that they are being studied.[24] We will seek to minimise the impact by informing staff that they are being observed and we wish them to behave as normal.[25]

Hospital staff interviews and surveys

Thirty hospital staff across six sites will be interviewed. Staff members will include those involved in delivering MaTI, such as heart failure specialist nurses, ward pharmacists, pharmacy technicians, cardiology ward nurses and site co-ordinators. We will identify and recruit secondary care staff through a combination of two approaches: (i) we will use information obtained during our ward observations about staff roles in delivering the intervention and approach staff directly; and (ii) we will also liaise with the appointed site co-ordinator to identify staff that are directly involved in the delivery of MaTI.

We will recruit hospital staff for surveys, aiming to recruit as many staff as possible involved in delivering MaTI. We will identify hospital staff with assistance from the site coordinators and seek informed consent from all staff.

For peer review only

Community pharmacist interviews and surveys

We will identify and recruit ten community pharmacists for interviews. Community pharmacists contacted through nominated pharmacies in each of the six sites will be interviewed. We will identity through the following two methods: the patient checklist forms (collected by the trials unit) containing details of the pharmacy used by patients in the trial; directly contacting and inviting community pharmacists to participate.

Surveys will be undertaken with community pharmacists involved in delivering the intervention. The number of surveys returned will be dependent on the number of differing community pharmacies patients have used as well as return rates. We will identify community pharmacists for surveys through the methods highlighted above for interviews.

Heart failure nurse surveys

We will recruit two to three community heart failure nurses in each of the six evaluation clusters to complete surveys. Community heart failure nurses will be identified through referrals made to the community heart failure services from the hospitals.

For all interviews and surveys, participants will be provided with an information sheet and informed, written consent will be obtained.

Data collection

Patient interviews

Semi-structured interviews with patients will be undertaken in patients' homes three months post-registration into the trial and will last approximately 45 minutes. Interviews, with patients and staff, will be audio recorded and transcribed verbatim. In the first site, patient data will be collected in a block of three to four relatively early in the implementation of the intervention (in the first three months). In subsequent sites, we may sample during middle and late phases of implementation which will be determined based on the data collected. As the trial progresses, we will become more familiar with how the intervention is being implemented. Interview schedules will include a range of questions, probes and prompts and will explore patient experiences of the intervention components.

Observations of intervention wards

Two researchers (CP/HI) will conduct unstructured and structured observations of clinical staff (heart failure nurses, cardiology nurses, cardiology pharmacists) of adherence to the intervention (content, coverage, frequency and duration). These observations will take place in hospital six months post-registration of the first patient recruited at that site, focusing in-depth on the discharge process and introduction of the MaTI toolkit. Observations will be two and a half hours per site designed to

For peer review only

capture not only delivery of the MaTI (structured) but also to augment our understanding of the hospital ward culture and environment (unstructured). Focused and general observation data will be collected through the use of a designed structured observation tool and field notes.

The focus of the observations will be on the interaction between staff and patients, particularly around the discharge process and completion/use of the toolkit. Other aspects of the intervention e.g. transfer of information, time spent with patients and level of patient understanding will be observed if possible. We will collect quantitative data using structured observations to record actions or behaviours, for example, how information was introduced and provided to patients. The structured observation may pose a greater challenge to collect as it will not be possible to identify in advance when MaTI related activities, such as the introduction of the toolkit, will occur. We will liaise with staff within the site to help us identify suitable times to conduct this structured observation. In the unstructured observations we will seek to develop a more general understanding of the ward culture and the ways the staff interact with patients and each other in the delivery of care. Unstructured data will be collated through field notes.

Hospital staff interviews and surveys

Semi-structured interviews with hospital staff will be conducted using an interview schedule covering staff experiences in delivering the intervention. These will take place at the hospital and at the end of site trial implementation i.e. two weeks post-discharge of the last recruited patient to avoid influencing intervention implementation during the trial. Survey data will be collected through paper questionnaires provided to staff when visiting the hospital at the end of site implementation.

Community pharmacist interviews and surveys

Community pharmacy semi-structured interviews will focus on how the pharmacists interacted with the enhanced communication from the hospital and how they engaged with patients post-discharge. An interview schedule will be used to collect data on the interventions perceived usability and impact. These will take place at the end of site implementation i.e. two months post-discharge of the last recruited patient. Interviews may take place face to face or via phone. Survey data will be collected through questionnaires to staff via post once the intervention has finished at that site.

Heart failure nurse surveys

Heart failure nurses will be invited to complete postal surveys at the end of site implementation. Table 1 illustrates the timing of all data collection.

Table 1

**Data collection across six process evaluation sites***

For peer review only

**Interview Patient**	**Interview Hospital staff**	**Interview Community Pharmacists**	**Focused and general observations**	**Survey hospital staff**	**Survey Community Pharmacists**	**Survey Community Heart Failure Nurses**
20 in total	30 in total	10 in total	2.5 hours	Sampling frame is all hospital staff involved in delivery of the intervention	Sampling frame is all community pharmacists that have delivered the intervention	12 approx.
3 months post patient registration into trial	2 weeks post discharge last patient	2 months post discharge last patient	6 months post implementation	2 weeks post discharge last patient	2 months post discharge last patient	2 months post discharge last patient

* Within each site, data collection will be sequential. Across the six sites, data collection will be non-sequential as it will depend on recruitment rates at each site.

Analysis

The qualitative analysis will be undertaken using a two-step process:

Step 1 Framework analysis

The process of interpreting the transcripts will take place whilst interviews are still being conducted. This will give the research team the opportunity to explore emerging themes in detail in subsequent interviews. The interviews and unstructured observations will be analysed using the Framework approach, which involves detailed familiarisation with the data, identifying key themes, interpreting the findings within the context of similar research studies, and considering policy and practice.[26] The emerging analysis will be thematic and iterative with regular discussions taking place with the process evaluation team. This involvement will support our interpretation of the interview data. The analysis will be theoretically informed by COM-B[22] and AMO.[20-22]

Step 2 Consolidated Framework for Implementation Research (CFIR)

Following initial theme generation, we will review the data using the Consolidated Framework for Implementation Research (CFIR).[18] The analysis will involve mapping barriers and facilitators onto domains within CFIR.

Quantitative data will include survey, structured observations and data on adherence to the intervention. The survey data will be entered into secure databases. Descriptive statistics will be employed to analyse survey data. Data relating to adherence to MaTI is being collected from all

For peer review only

intervention sites. This consists of checklists detailing which components of the intervention were implemented for each patient. We will use these data to inform and explain the findings in the process evaluation sites.

Additional data

Where appropriate, we may consider using the additional data (collected for all sites as part of the wider trial) to inform, explain and triangulate findings with the process evaluation. For example, we may decide that we need more structured information on completion of the MaTI checklist to consider alongside our staff interviews and so clarify which steps of the MaTI different staff members carried out.

Additional data sources include:

- Site Feasibility Questionnaire (initial questions sent to sites to assess their suitability to take part in the trial covering areas such as clinical pathways, staffing levels/ types of specialist staff, communication with community pharmacy and the number of patients)

- Patient Experience Survey (completed by patients at two weeks/ six weeks/ twelve months post discharge)

- MaTI checklist completed in the hospital (monitors adherence to the main components of the intervention)

- National Heart Failure Audit Data[27] (reports on the characteristics of patients admitted with acute or sub-acute heart failure, in-hospital investigations and care, treatment given and the discharge planning and follow-up)

- Community pharmacy data collection form (describes implementation within community pharmacy)

Analysis will be integrative in order to clarify and explain the predominant systems and their implications; qualitative and quantitative data will be consolidated through a process of 'parallel mixed analysis'.[28] This includes an independent analysis of the quantitative and qualitative data to provide an understanding of key phenomena and the two understandings will be integrated using meta-inferences. For example, key findings will be generated iteratively, explicitly supported by quantitative data (such as structured observational and survey data) and substantiated or augmented by thematic qualitative data (such as interview data and field notes), and vice-versa. Survey data will be compared across the six clusters to identify any differences in staff perceptions of the barriers and facilitators to delivery. Analysis will be descriptive.

For peer review only

The combined analysis will therefore meet our key aims to inform interpretation of the trial findings, inform implementation of the intervention on a wider scale (for example, other long-term conditions) and aid potential future implementation of the intervention. This will be achieved by providing an in depth understanding of the overall implementation, mechanisms of impact and external factors (infra structure) that may influence delivery and functioning of the intervention.

Patient and public involvement

The ISCOMAT study has a patient-led steering group that is involved in all stages of the research process, including the process evaluation. Due to the process evaluation's iterative design we will regularly consult with the group via meetings and phone/email. The group will continue to be consulted on the research design, questions, outcome measures and findings. In particular the members of the group contribute to reviews and evaluations, as well as reading and considering study and consultation documents from a patient perspective. The group's expertise through their experiences of living with heart failure will be crucial in understanding patients' experiences with the MaTI intervention in particular.

ETHICS AND DISSEMINATION

The process evaluation has been approved as part of the ISCOMAT trial by the Research Ethics Committee and the UK Health Research Authority REC: 18/YH/0017 / IRAS: 231431.

Findings will be disseminated via academic and policy conferences, peer reviewed publications, social media e.g. Twitter, with further avenues for dissemination to be agreed upon with our patient led steering group.

CONCLUSION

In this paper we have described the design and methods for the mixed-methods process evaluation of the NIHR funded ISCOMAT cluster randomised controlled trial which will test the effectiveness of a complex behavioural intervention aimed at improving medications management at the interface between hospital and community for patients with hospitalised with heart failure. This process evaluation protocol demonstrates the importance of process evaluations for understanding outcomes in the clinical trial, as well as providing guidance for future process evaluations. We have followed the Medical Research Council (MRC) recommendations and guidance on the delivery of process evaluation[13] in order to support the standardisation of process evaluations.

Acknowledgements

For peer review only

We would like to thank Alison Blenkinsopp, Gerry Armitage, Lauren Moureau, Jan Speechley, the ISCOMAT Patient-led Steering Group, the ISCOMAT Trial Management Group, Trial Steering Committee and the Programme Steering Committee.

Competing interests statement

The authors are not aware of any competing interests.

Funding

This study was supported by the National Institute of Health Research programme Improving the safety and continuity of medicines management at care transitions (ISCOMAT) RP-PG-0514-20009.

Disclaimer

The views expressed are those of the authors and not necessarily those of the NHS, the NIHR or the Department of Health and Social Care.

Authors' contributions

CP, LB, BF, HI, SA, CPG, PG, AF and DPA developed the detail of the process evaluation protocol. CP drafted the manuscript and all authors reviewed it critically for intellectual content and approved the final version submitted for publication.

Our collaborators include members of the wider ISCOMAT Programme Management Team who contributed to previous work packages and the ongoing programme including: Jon Silcock, David K. Raynor, Robert Turner, John Wright, Ian Kellar, Roberta Longo, Ivana Holloway, Chris Bojke, Leeds Clinical Trials Research Unit.

REFERENCES

1. Dewan P, Rørth R, Jhund PS, et al. Income inequality and outcomes in heart failure: a global between-country analysis. *JACC: Heart Failure*. 2019;7(4):336-46.doi:10.1016/j.jchf.2018.11.005
2. British Heart Foundation. UK Factsheet 2020 [Available from: https://www.bhf.org.uk/what-we-do/our-research/heart-statistics. Date accessed 14/04/2020
3. Conrad N, Judge A, Tran J, et al. Temporal trends and patterns in heart failure incidence: a population-based study of 4 million individuals. *The Lancet*. 2018;391(10120):572-80.doi:10.1016/S0140-6736(17)32520-5
4. Gho JMIH, Schmidt AF, Pasea L, et al. An electronic health records cohort study on heart failure following myocardial infarction in England: incidence and predictors. *BMJ open*. 2018;8(3):e018331.doi: 10.1136/bmjopen-2017-018331
5. National Institute for Health and Social Care Excellence. Chronic heart failure in adults: diagnosis and management NICE guideline [NG106] 2018 [Available from: https://www.nice.org.uk/guidance/ng106. Date accessed 14/04/2020

6. Phipps DL, Morris RL, Blakeman T, et al. What is involved in medicines management across care boundaries? A qualitative study of healthcare practitioners' experiences in the case of acute kidney injury. *BMJ open*. 2017;7(1):e011765.doi: 10.1136/bmjopen-2016-011765
7. Urban R, Paloumpi E, Rana N, et al. Communicating medication changes to community pharmacy post-discharge: the good, the bad, and the improvements. *Int J Clin Phar,*. 2013;35(5):813-20.doi: 10.1007/s11096-013-9813-9
8. Fylan B, Marques I, Ismail H, et al. Gaps, traps, bridges and props: a mixed-methods study of resilience in the medicines management system for patients with heart failure at hospital discharge. *BMJ Open*. 2019;9(2):e023440.10.1136/bmjopen-2018-023440
9. Inamdar AA, Inamdar AC. Heart failure: diagnosis, management and utilization. *Journal of clinical medicine*. 2016;5(7):62.doi: 10.3390/jcm5070062
10. Van Spall HGC, Lee SF, Xie F, et al. Effect of patient-centered transitional care services on clinical outcomes in patients hospitalized for heart failure: the PACT-HF randomized clinical trial. *Jama*. 2019;321(8):753-61.doi: 10.1001/jama.2019.0710
11. Raynor DK, Ismail H, Blenkinsopp A, et al. Experience-based co-design—Adapting the method for a researcher-initiated study in a multi-site setting. *Health Expectations*. 2020.doi:https://doi.org/10.1111/hex.13028
12. Ellard DR, Taylor SJC, Parsons S, et al. The OPERA trial: a protocol for the process evaluation of a randomised trial of an exercise intervention for older people in residential and nursing accommodation. *Trials*. 2011;12(1):28.doi: 10.1186/1745-6215-12-28
13. Moore GF, Audrey S, Barker M, et al. Process evaluation of complex interventions: Medical Research Council guidance. *British Medical Journal*. 2015;350:h1258.doi:https://doi.org/10.1136/bmj.h1258
14. Mann C, Shaw A, Guthrie B, et al. Protocol for a process evaluation of a cluster randomised controlled trial to improve management of multimorbidity in general practice: the 3D study. *BMJ open*. 2016;6(5):e011260.doi:http://dx.doi.org/10.1136/bmjopen-2016-011260
15. Grant A, Treweek S, Dreischulte T, et al. Process evaluations for cluster-randomised trials of complex interventions: a proposed framework for design and reporting. *Trials*. 2013;14(1):15.doi:https://doi.org/10.1186/1745-6215-14-15
16. Ellard DR, Thorogood M, Underwood M, et al. Whole home exercise intervention for depression in older care home residents (the OPERA study): a process evaluation. *BMC Med*. 2014;12(1):1
17. Sheard L, O'Hara J, Armitage G, et al. Evaluating the PRASE patient safety intervention-a multi-centre, cluster trial with a qualitative process evaluation: study protocol for a randomised controlled trial. *Trials*. 2014;15(1):2282. doi:10.1186/1745-6215-15-420
18. Damschroder L, Aron D, Keith R. SR K, Alexander J, Lowery J. Fostering implementation of health services research findings into practice: a consolidated framework for advancing implementation science. *Implement Sci*. 2009;4:50
19. Keith RE, Crosson JC, O'Malley AS, et al. Using the Consolidated Framework for Implementation Research (CFIR) to produce actionable findings: a rapid-cycle evaluation approach to improving implementation. *Implementation Science*. 2017;12(1):15.doi:10.1186/s13012-017-0550-7
20. Macky K, Boxall P. The relationship between 'high-performance work practices' and employee attitudes: an investigation of additive and interaction effects. *The International Journal of Human Resource Management*. 2007;18(4):537-67.doi:10.1080/09585190601178745
21. Purcell J. Understanding the people and performance link: Unlocking the black box: CIPD Publishing; 2003.
22. Michie S, Van Stralen MM, West R. The behaviour change wheel: a new method for characterising and designing behaviour change interventions. *Implementation science*. 2011;6(1):42
23. Coleman EA, Mahoney E, Parry C. Assessing the quality of preparation for posthospital care from the patient's perspective: the care transitions measure. *Medical care*. 2005:246-55.doi:10.1097/00005650-200503000-00007

For peer review only

24. Roethlisberger F, Dickson W. Management and the Worker Cambridge, MA: Harvard University Press; 1939.
25. Campbell JP, Maxey VA, Watson WA. Hawthorne effect: implications for prehospital research. *Annals of emergency medicine*. 1995;26(5):590-4
26. Ritchie J, Lewis J, Nicholls CM, et al. Qualitative research practice: A guide for social science students and researchers: Sage; 2013.
27. National Institute for Cardiovascular Outcomes Research (NICOR). National Heart Failure Audit 2017/18 2017/18 [Available from: https://www.nicor.org.uk/national-cardiac-audit-programme/heart-failure-heart-failure-audit/. Date accessed 25/11/2019
28. Creswell JW, Clark VLP. Designing and conducting mixed methods research: Sage publications; 2017.

For peer review only

Medical Research Council process evaluation checklist[1]

Checklist	Response	Where in protocol?
**Working with policy and practice stakeholders**		
1. Are there any potential conflicts of interest arising from the relationship between evaluators and policy/practice stakeholders?	No	Page 12
2. Have the authors described how they will address these and ensure that the evaluation remains independent?	N/A	N/A
3. Does the proposal set out a clear plan for communicating findings to policy and practice stakeholders during the evaluation?	Yes	Ethics and dissemination on page 11
**Relationships between evaluation components**		
4. Is the relationship between the process evaluation and other evaluation components clearly defined and justified?	To an extent. More detail to come in results paper	'The process evaluation' on page 4
5. Will process and outcomes evaluation be conducted by the same team or by separate teams?	Separate teams	'The process evaluation' on page 4
6. If the former, how will researchers ensure that knowledge of outcomes or process does not bias analysis of the other?	N/A	N/A
7. If the latter, is there clear oversight of the two components?	Yes	'Process evaluation' on page 4 (regular meetings)
8. Is it clear that the principal investigator values all aspects of the evaluation, and will provide effective oversight of all aspects of the evaluation?	Yes	'Process evaluation' on page 4 (regular meetings)
**Intervention description and theory**		
9. Is the intended intervention fully described?	No	'Study design' on page 5 indicates that we can only provide limited information
10. Are standardised terminology and definitions of intervention components adopted where possible?	As above	As above
11. Are the structures and processes involved in intervention delivery fully described?	As above	As above
12. If appropriate, will a full intervention manual be made publicly available?	Yes if intervention is successful	N/A for protocol paper
13. Is a clear, plausible, set of causal assumptions specified and justified (for example, in a logic model)?	To be described in a separate paper	N/A
14. Does this draw upon appropriate	Yes	Page 5 'Study design' AMO, COM-B

theories?		and CFIR
15. If not, are there plans to develop a theory as part of the research?	N/A	N/A
16. Have the authors planned to review these assumptions with policy and practice stakeholders to explore agreement and divergence on what the intervention is, and how it will work?	Yes	Completed and discussed in another paper
**Process evaluation aims and research questions**		
17. Are the research questions clear, important and well justified with reference to the theory of the intervention and the status of the evidence base? What decisions will they inform?	Yes	Research questions and objectives on page 4. Justified on page 3 in 'Introduction'.
18. Have the authors considered whether previous process evaluations have been conducted of interventions involving similar components or theories of change?	Yes	Literature highlighted in 'Study design' on page 5
19. Have they adopted comparable aims and methods, or justified not doing so?	Yes	Methods outlined from page 5
20. Has the theory of the intervention (or logic model) been used to identify key areas of uncertainty for investigation by process evaluation?	Yes	Yes CFIR AMO and COM-B will be included in data collection and analysis processes as highlighted on page 5
21. Have the authors considered which components may prove most challenging to implement (e.g. which represent more fundamental change, or for which there is least agreement on what they are and the purposes they serve)?	Yes	Detail to be provided in results paper
22. Have the authors considered for which causal assumptions evidence is most equivocal?	Yes	To be reported in results paper
23. How will unanticipated consequences be captured?	Yes	To be reported in results paper. Changes from protocol will be recorded in a table
24. Is there linkage between research aims? Do they fit together to address the overall study aim?	Yes	Study aims to be found on page 4
25. If conducted alongside an outcomes evaluation, is the added value of the process evaluation explained? Is it clear how the research will enhance the interpretation of outcomes?	Yes	Explained in 'Study design' page 5
26. Will process evaluation provide sufficient assurances regarding the internal validity of the outcomes	Yes	Examining data from the trial, e.g. Hospital (MaTI) checklist (monitors adherence to the main components of

For peer review only

evaluation?		the intervention) Page 10
27. Will it enable policymakers/practitioners to understand how the intervention might be applied in different contexts?	N/A protocol paper	To be reported in results paper
28. Have the authors stated how and when they will combine process and outcomes data?	N/A protocol paper	To be reported in results paper
**Selection of methods to address research questions**		
29. Are the quantitative and qualitative methods selected appropriate to the research questions?	Yes	Pages 5 to 11 discuss methods used. More detail will be provided in results paper
30. Will implementation be captured in sufficient detail to establish consistency with the theory of the intervention?	N/A protocol paper	To be reported in results paper
31. Are existing validated measures used where possible? Are plans to validate new measures included?	Yes	Page 9 on framework analysis and CFIR
32. How will emerging changes, adaptations or additions to the intervention be captured?	N/A protocol paper	N/A
33. Are the quantitative methods appropriate? (e.g. ‘tick box’ self-report by implementers of intervention delivery should be avoided if possible).	Yes	Page 6-11 we describe quantitative methods
34. Are the qualitative methods appropriate?	Yes	Pages 6-11 we describe qualitative methods
35. Have the authors considered how change in practice as a result of being observed or measured will be minimised?	Yes	Page 6 on hawthorn effect
36. Have the authors considered the timing of data collection, and its impact on the data collected?	Yes	Page 8-9 on timing of data collection
37. Have the authors investigated whether any routine programme monitoring data can be used? If so, are there plans to check their validity and reliability?	Yes	Interviews will explore routine data such as MaTI checklists to explain if there are poor quality results. Page 11
38. Have the authors stated how quantitative and qualitative methods will be combined?	Yes	Page 10-11 Greater detail will be given in results paper
39. Have the authors considered how they will respond if challenges emerge during the evaluation - for example, if serious implementation failures are identified which need deeper investigation?	Yes	We describe possible challenges carrying out observations and how this will be addressed. Page 6
**Resource considerations in collecting/analysing process data**		
40. Who will lead or conduct the process evaluation? Do they have,	Yes	The lead is named on the paper

or have direct access to, appropriate expertise and experience?		
41. Does the research team have sufficient expertise in quantitative and qualitative methods and relevant social science theory?	Yes	N/A
42. Is sufficient time, funding and staff resource included for data collection, analysis (including sufficient time to conduct good quality analysis of qualitative data, with quality checks by a second coder where appropriate) and reporting?	Yes	Considered regularly at meetings
**Analysis and reporting**		
43. Has consideration been given to the use of quantitative process measures for modelling variations in outcomes and/or cost-effectiveness?	Yes	Separate cost evaluation is being conducted
44. Is the relationship between qualitative data components and outcomes and/or cost effectiveness analysis clear?	Yes	Relationship between qualitative data and outcomes discussed on page 11. Separate cost evaluation is being conducted.
45. Is there a coherent strategy for dissemination to an academic audience and wider stakeholders?	Yes	Page 11. Discussed at regular meeting.

1. Moore GF, Audrey S, Barker M, et al. Process evaluation of complex interventions: Medical Research Council guidance. *British Medical Journal*. 2015;350:h1258.doi:https://doi.org/10.1136/bmj.h1258

For peer review only

BMJ Open

Improving the Safety and Continuity Of Medicines management at Transitions of care (ISCOMAT): protocol for a process evaluation of a cluster randomised control trial

Journal:	*BMJ Open*
Manuscript ID	bmjopen-2020-040493.R1
Article Type:	Protocol
Date Submitted by the Author:	14-Oct-2020

Complete List of Authors:	Powell, Catherine; University of Bradford Faculty of Life Sciences, School of Pharmacy and Medical Sciences; Wolfson Centre for Applied Health Research<br>Breen, Liz; University of Bradford Faculty of Life Sciences, School of Pharmacy and Medical Sciences; NIHR Yorkshire and Humber Patient Safety Translational Research Centre<br>Fylan, Beth; University of Bradford Faculty of Life Sciences, School of Pharmacy and Medical Sciences; NIHR Yorkshire and Humber Patient Safety Translational Research Centre<br>Ismail, Hanif; University of Bradford Faculty of Life Sciences, School of Pharmacy and Medical Sciences; Wolfson Centre for Applied Health Research<br>Alderson, Sarah; University of Leeds School of Medicine<br>Gale, Chris; University of Leeds, Leeds Institute of Cardiovascular and Metabolic Medicine<br>Gardner, Peter; University of Bradford Faculty of Life Sciences, School of Pharmacy and Life Sciences; Wolfson Centre for Applied Health Research<br>Farrin, Amanda; University of Leeds, Clinical Trials Research Unit<br>Alldred, David ; University of Leeds, School of Healthcare; NIHR Yorkshire and Humber Patient Safety Translational Research Centre

**Primary Subject Heading**:	Cardiovascular medicine
Secondary Subject Heading:	Research methods
Keywords:	Heart failure < CARDIOLOGY, CARDIOLOGY, Clinical trials < THERAPEUTICS, STATISTICS & RESEARCH METHODS

SCHOLARONE™
Manuscripts

I, the Submitting Author has the right to grant and does grant on behalf of all authors of the Work (as defined in the below author licence), an exclusive licence and/or a non-exclusive licence for contributions from authors who are: i) UK Crown employees; ii) where BMJ has agreed a CC-BY licence shall apply, and/or iii) in accordance with the terms applicable for US Federal Government officers or employees acting as part of their official duties; on a worldwide, perpetual, irrevocable, royalty-free basis to BMJ Publishing Group Ltd ("BMJ") its licensees and where the relevant Journal is co-owned by BMJ to the co-owners of the Journal, to publish the Work in this journal and any other BMJ products and to exploit all rights, as set out in our licence.

The Submitting Author accepts and understands that any supply made under these terms is made by BMJ to the Submitting Author unless you are acting as an employee on behalf of your employer or a postgraduate student of an affiliated institution which is paying any applicable article publishing charge ("APC") for Open Access articles. Where the Submitting Author wishes to make the Work available on an Open Access basis (and intends to pay the relevant APC), the terms of reuse of such Open Access shall be governed by a Creative Commons licence – details of these licences and which Creative Commons licence will apply to this Work are set out in our licence referred to above.

Other than as permitted in any relevant BMJ Author's Self Archiving Policies, I confirm this Work has not been accepted for publication elsewhere, is not being considered for publication elsewhere and does not duplicate material already published. I confirm all authors consent to publication of this Work and authorise the granting of this licence.

For peer review only

Improving the Safety and Continuity Of Medicines management at Transitions of care (ISCOMAT): protocol for a process evaluation of a cluster randomised control trial

Corresponding author

Dr Catherine Powell

Room M24

Richmond Building

School of Pharmacy and Medical Sciences,

University of Bradford,

Richmond Rd, Bradford BD7 1DP, UK

c.powell2@bradford.ac.uk

01274 236338

Authors

Dr Catherine Powell, School of Pharmacy and Medical Sciences, University of Bradford, Richmond Road, Bradford, BD7 1DP, UK. Wolfson Centre for Applied Health Research, Bradford, UK. https://orcid.org/0000-0001-7590-0247

Dr Liz Breen, School of Pharmacy and Medical Sciences, University of Bradford, Richmond Road, Bradford, BD7 1DP, UK. NIHR Yorkshire and Humber Patient Safety Translational Research Centre. Bradford Institute for Health Research, Temple Bank House, Bradford, BD9 6RJ. Wolfson Centre for Applied Health Research, Bradford, UK. https://orcid.org/0000-0001-5204-1187

Dr Beth Fylan, School of Pharmacy and Medical Sciences, University of Bradford, Richmond Road, Bradford, BD7 1DP, UK. NIHR Yorkshire and Humber Patient Safety Translational Research Centre. Bradford Institute for Health Research, Temple Bank House, Bradford, BD9 6RJ, UK. Wolfson Centre for Applied Health Research, Bradford, UK. https://orcid.org/0000-0003-0599-4537

Dr Hanif Ismail, School of Pharmacy and Medical Sciences, University of Bradford, Richmond Road, Bradford, BD7 1DP, UK. Wolfson Centre for Applied Health Research, Bradford, UK. https://orcid.org/0000-0002-7885-6648

Dr Sarah Alderson, School of Medicine, Worsley Building, University of Leeds, Leeds LS2 9JT, UK. https://orcid.org/0000-0002-5418-0495

Professor Chris P Gale, Leeds Institute of Cardiovascular and Metabolic Medicine, University of Leeds, Leeds, UK. https://orcid.org/0000-0003-4732-382X

Professor Peter Gardner, School of Pharmacy and Medical Sciences, University of Bradford, Richmond Road, Bradford, BD7 1DP, UK. Wolfson Centre for Applied Health Research, Bradford, UK. https://orcid.org/0000-0002-8799-0443

Professor Amanda J Farrin, Clinical Trials Research Unit, Leeds Institute of Clinical Trials Research, University of Leeds, Leeds, LS2 9JT, UK. http://orcid.org/ 0000-0002-2876-0584

For peer review only

Professor David P Alldred, University of Leeds, School of Healthcare, Baines Wing, Leeds LS2 9DA, UK. NIHR Yorkshire and Humber Patient Safety Translational Research Centre. Bradford Institute for Health Research, Temple Bank House, Bradford, BD9 6RJ, UK. Wolfson Centre for Applied Health Research, Bradford, UK. https://orcid.org/0000-0002-2525-4854

ISCOMAT Programme Management Team

Word count (excluding title page, abstract, references, figures and tables): 3694

Key words: Heart failure, Cardiology, Clinical trials, Statistics and Research Methods

ABSTRACT

Introduction: A key priority for the UK National Health Service (NHS) and patients is to ensure that medicines are used safely and effectively. However, medication changes are not always optimally communicated and implemented when patients transfer from hospital into community settings. Heart failure is a common reason for admission to hospital. Patients with heart failure have a high burden of morbidity, mortality and complex pharmacotherapeutic regimes. The Improving the Safety and Continuity Of Medicines management at Transitions of care (ISCOMAT) programme comprises a cluster randomised controlled trial which will test the effectiveness of a complex behavioural intervention aimed at improving medications management at the interface between hospitals discharge and community care. We will conduct a rigorous process evaluation to inform interpretation of the trial findings, inform implementation of the intervention on a wider scale and aid dissemination of the intervention.

Methods and analysis: The process evaluation will be conducted in six purposively selected intervention sites (i.e. hospital trusts and associated community pharmacies) using a mixed-methods design. Fidelity and barriers/enablers of implementation of the Medicines at Transitions Intervention (MaTI) will be explored using observation, interviews (20 patients, 40 healthcare professionals), surveys and routine trial data collection on adherence to MaTI. A parallel mixed analysis will be applied. Qualitative data will be thematically analysed using Framework analysis and survey data will be analysed descriptively. Data will be synthesised, triangulated and mapped to the Consolidated Framework for Implementation Research (CFIR) where appropriate. The process evaluation commenced on June 2018 and is due to end on February 2021.

Ethics and dissemination: Approved by Research Ethics Committee and the UK Health Research Authority REC: 18/YH/0017 / IRAS: 231431. Findings will be disseminated via academic and policy conferences, peer-reviewed publications and social media.

Trial registration number: ISRCTN: 66212970/

For peer review only

ARTICLE SUMMARY

Strengths and limitations of this study

- An evaluation of a cluster randomised controlled trial of a complex behavioural intervention for a high priority healthcare issue.
- Views of patients and health professionals working across both primary and secondary care.
- A key strength is our mixed methods and the flexibility of our approach in light of emerging issues from the study and the shifts in healthcare practice and policy.
- The process evaluation is limited to six purposively selected intervention sites.

INTRODUCTION

Heart failure presents a major challenge to healthcare systems globally.[1] In the UK it is thought to affect the lives of over 920,000 people.[2] In a study of four million individuals, heart failure cases have been found to increase 12% from 2002 to 2014.[3] Myocardial infarction has been identified as a risk for heart failure.[4] Heart failure is treated by a combination of medication, lifestyle changes and interventional surgery depending on the severity of the condition. Typically, the pharmacological treatment pathway involves a combination of medicines that are titrated to the optimal level that patients can tolerate. If not managed well (or if the patient has not been diagnosed and treated in primary care) they may be admitted to hospital for treatment and to be stabilised. Approximately 5% of all emergency hospital admissions are for heart failure.[5] Ongoing treatment plans following discharge from hospital may not be fully implemented due to failures in communication between healthcare providers or a lack of specialist staff in the community.[6, 7] Patients may not understand their medicines, what they are for and why they need to take them because, for example, medical language may create a gap in patients' understanding.[8] Patients may therefore deteriorate, which can result in a re-admission to hospital or death.[9]

The ISCOMAT study

ISCOMAT is a five-year National Institute for Health Research (NIHR) funded research programme, which aims to optimise the way heart failure patients are supported with their medicines when they move from hospital to home. This may contribute to improving patients' health through helping them better understand and use their medicines. ISCOMAT also aims to improve the way health professionals work together in order to improve communication and optimise medicines use when patients return home. Similar international studies have examined patient-centred care transitions for patients hospitalized for heart failure and found clinical outcomes did not improve. ISCOMAT is designed for the UK health system and may show improvements in clinical outcomes in this setting.[10] In earlier work packages, we explored the resilience of the medicines management

For peer review only

pathway for heart failure and then used Experienced Based Co-Design to develop the Medicines at Transitions Intervention (MaTI) with patients and professionals.[8, 11] The MaTI was designed to make best use of medicines and reduce harm through: the provision of information to the patient; enhanced communication between hospital and the patient's community pharmacist; and increased engagement of the community pharmacist post discharge. We have provided limited information about the intervention here to avoid potential contamination of the ongoing trial. A more detailed description of the MaTI intervention will be published alongside feasibility testing. The cluster randomised controlled trial is testing the effectiveness of the MaTI. Patients will be recruited from cardiology wards in 42 acute NHS trusts across England, over approximately 12 months. The recruitment target is 50 patients from each cardiology ward (target n=2100 patients in total). Sites are randomised to either treatment as usual or to the MaTI. A site co-ordinator is responsible for organising the trial and implementation of the MaTI at each site.

The process evaluation

Process evaluations are increasingly carried out alongside randomised controlled trials.[12, 13] They are particularly well suited to trials of complex interventions in multiple sites where the intervention may be implemented differently throughout sites and help us to understand more about the practical problems encountered and how they were resolved. We will follow the Medical Research Council (MRC) recommendations and guidance on process evaluations.[13] The process evaluation will examine the effect of organisational context and setting on intervention delivery, and how the intervention is best implemented. There are no predefined methods that a process evaluation must adopt although they typically involve mixed methods.[14, 15] Although the process and outcomes evaluation will be conducted by separate teams, the trial and process evaluation teams will meet regularly to have oversight.

Aims and objectives

The aims of the process evaluation are to inform interpretation of the trial findings, inform implementation of the intervention on a wider scale (for example, other long term conditions) and aid potential future implementation of the intervention.

Our objectives at each of the six process evaluation sites are to:

- Determine the degree to which the intervention is delivered (internal fidelity)
- Explore and explain the relationship between intervention implementation and the trial outcomes
- Identify barriers and facilitators for the successful implementation and roll out of the intervention (should the intervention be effective)

For peer review only

PROCESS EVALUATION METHODS

Study design

In addition to the aforementioned MRC guidance,[13] we will draw on other relevant literature,[16, 17] in particular the Consolidated Framework for Implementation Research (CFIR).[18] The CFIR is a conceptual framework that was developed to guide systematic assessment of multilevel implementation contexts to identify factors that might influence intervention implementation and effectiveness.[19] The MaTI intervention is implemented at multiple levels at each site including trusts, secondary and primary health care professionals and patients. The CFIR framework generates a knowledge base for implementation across multiple settings within five major domains: intervention characteristics, inner setting, outer setting, individual characteristics, and implementation process. Domains will be explored and additional relevant theoretical frameworks applied as appropriate. We will also draw on human resource management evaluation, specifically the ability, motivation and opportunity (AMO) model,[20, 21] and capability, opportunity, and motivation (COM-B) model.[22] These models are complementary and highlight how policy interventions that require changes in staff behaviour are shaped by their ability to work in different ways, in terms of skills, their level of motivation and their opportunities to change practice.

The study design is a parallel mixed synthesis study using quantitative and qualitative data from six intervention sites of the total 42 sites in the cluster randomised controlled trial. Methods will involve non-participant observations, semi-structured interviews and surveys. In order to capture data on barriers and facilitators to implementation we will collect data from health professionals working along the patient pathway (from both secondary and primary care), patients and community pharmacists. We will also utilise fidelity data on adherence to MaTI that is being collected within each intervention site in the trial. We will explore and explain the relationship between intervention implementation and the trial outcomes in the six process evaluation sites through analysing secondary outcome data. These data will indicate whether the intervention improves patient understanding of their medicines and satisfaction with medicines-related care at two and six weeks post-discharge and twelve months post-registration from the Patient Experience Survey (PES) (a validated item from Coleman's transition measure [23]), alongside observations and implementation data collected as part of the process evaluation. Process evaluations need to be flexible in the context of the ongoing trials they are evaluating and we will record and describe adaptations to this protocol when the findings are published.

Sampling and recruitment

We aim to purposively sample six intervention sites based on the following criteria:

- University and non-university hospitals

For peer review only

- Method for transferring medicines discharge information to community pharmacists' e.g. electronic system, post, telephone
- Sites located across different geographic areas of England

Within each of the six selected sites we will recruit patients and staff for interviews and surveys. We will also conduct observations at each of these sites. The sampling approach will be iterative and following sampling of one-to-two pilot sites; consideration of the initial data in relation to fidelity will be made.

Patient interviews

Twenty patients in total will be sampled for interviews (three to four patients across each of the six sites). We will adopt a purposive sampling strategy to meet our target characteristics in terms of gender, age, length of diagnosis, and fidelity to intervention. After piloting, we may need to modify our approach to enable diversity in relation to our sampling criteria. Patients approached will be those that have participated in the trial and received the MaTI intervention.

Observations of intervention wards

We will seek permission to conduct non-participant observations of staff at ward level (two and a half hours per site). Staff members will be provided with information sheets and a script for informing patients of the researchers' presence. A maximum of two researchers will be present at any one time. Staff will have the opportunity to opt out of the observation. A potential limitation of conducting overt observation is the Hawthorne effect by which individuals being observed may alter their behaviour because they are aware that they are being studied.[24] We will seek to minimise the impact by informing staff that they are being observed and we wish them to behave as normal.[25]

Hospital staff interviews and surveys

Thirty hospital staff across six sites will be interviewed. Staff members will include those involved in delivering MaTI, such as heart failure specialist nurses, ward pharmacists, pharmacy technicians, cardiology ward nurses and site co-ordinators. We will identify and recruit secondary care staff through a combination of two approaches: (i) we will use information obtained during our ward observations about staff roles in delivering the intervention and approach staff directly; and (ii) we will also liaise with the appointed site co-ordinator to identify staff that are directly involved in the delivery of MaTI.

We will recruit hospital staff for surveys, aiming to recruit as many staff as possible involved in delivering MaTI. We will identify hospital staff with assistance from the site coordinators and seek informed consent from all staff.

For peer review only

Community pharmacist interviews and surveys

We will identify and recruit ten community pharmacists for interviews. Community pharmacists contacted through nominated pharmacies in each of the six sites will be interviewed. We will identity through the following two methods: the patient checklist forms (collected by the trials unit) containing details of the pharmacy used by patients in the trial; directly contacting and inviting community pharmacists to participate.

Surveys will be undertaken with community pharmacists involved in delivering the intervention. The number of surveys returned will be dependent on the number of differing community pharmacies patients have used as well as return rates. We will identify community pharmacists for surveys through the methods highlighted above for interviews.

Community heart failure nurse surveys

We will recruit up to five community heart failure nurses in each of the six evaluation clusters to complete surveys. A maximum of 30 surveys will be conducted in total. Community heart failure nurses will be identified through referrals made to the community heart failure services from the hospitals.

For all interviews and surveys, participants will be provided with an information sheet and informed, written consent will be obtained.

Data collection

Patient interviews

Semi-structured interviews with patients will be undertaken in patients' homes three months post-registration into the trial and will last approximately 45 minutes. Interviews, with patients and staff, will be audio recorded and transcribed verbatim. (This protocol was agreed prior to Covid-19. Data collection methods are being altered in response to the pandemic. For example, recent interviews are being conducted via phone. Our flexible approach to the protocol has facilitated these changes. Changes in methods will be tracked and reported as per pragmatic study development). In the first site, patient data will be collected in a block of three to four relatively early in the implementation of the intervention (in the first three months). In subsequent sites, we may sample during middle and late phases of implementation which will be determined based on the data collected. As the trial progresses, we will become more familiar with how the intervention is being implemented. Interview schedules will include a range of questions, probes and prompts and will explore patient experiences of the intervention components.

Observations of intervention wards

For peer review only

Two researchers (CP/HI) will conduct unstructured and structured observations of clinical staff (heart failure nurses, cardiology nurses, cardiology pharmacists) of adherence to the intervention (content, coverage, frequency and duration). These observations will take place in hospital six months post-registration of the first patient recruited at that site, focusing in-depth on the discharge process and introduction of the MaTI toolkit. Observations will be two and a half hours per site designed to capture not only delivery of the MaTI (structured) but also to augment our understanding of the hospital ward culture and environment (unstructured). Focused and general observation data will be collected through the use of a designed structured observation tool and field notes.

The focus of the observations will be on the interaction between staff and patients, particularly around the discharge process and completion/use of the toolkit. Other aspects of the intervention e.g. transfer of information, time spent with patients and level of patient understanding will be observed if possible. We will collect quantitative data using structured observations to record actions or behaviours, for example, how information was introduced and provided to patients. The structured observation may pose a greater challenge to collect as it will not be possible to identify in advance when MaTI related activities, such as the introduction of the toolkit, will occur. The two-and-a-half-hour period of observation will be discontinuous. We will wait at the site until relevant activity occurs, and we will liaise with staff within the site to help us identify suitable times to conduct this structured observation. Staff will help us identify when the intervention related activity is likely to occur. In the unstructured observations we will seek to develop a more general understanding of the ward culture and the ways the staff interact with patients and each other in the delivery of care. Unstructured data will be collated through field notes.

Hospital staff interviews and surveys

Semi-structured interviews with hospital staff will be conducted using an interview schedule covering staff experiences in delivering the intervention. These will take place at the hospital and at the end of site trial implementation i.e. two weeks post-discharge of the last recruited patient to avoid influencing intervention implementation during the trial. Survey data will be collected through paper questionnaires provided to staff when visiting the hospital at the end of site implementation. A maximum 180 surveys will be conducted.

Community pharmacist interviews and surveys

Community pharmacy semi-structured interviews will focus on how the pharmacists interacted with the enhanced communication from the hospital and how they engaged with patients post-discharge. An interview schedule will be used to collect data on the interventions perceived usability and impact. These will take place at the end of site implementation i.e. two months post-discharge of the last recruited patient. Interviews may take place face to face or via phone. Survey data will be collected

For peer review only

through questionnaires to staff via post once the intervention has finished at that site. A maximum 300 surveys will be conducted.

Community heart failure nurse surveys

Community heart failure nurses will be invited to complete postal surveys at the end of site implementation. A maximum 30 surveys will be conducted. We do not plan to interview community heart failure nurses because our primary focus is those delivering the intervention i.e. hospital staff and community pharmacists. However, the survey data will explore whether nurses used the toolkit with patients in the community, whether the toolkit enhanced patient care and whether nurses would advocate the use of the toolkit to support patient treatment. Table 1 illustrates the timing of all data collection.

Table 1 Data collection across six process evaluation sites*

Interview Patient	Interview Hospital staff	Interview Community Pharmacists	Focused and general observations	Survey hospital staff	Survey Community Pharmacists	Survey Community Heart Failure Nurses
20 in total	30 in total	10 in total	2.5 hours	180 max. Sampling frame is all hospital staff involved in delivery of the intervention	300 max. Sampling frame is all community pharmacists that have delivered the intervention	30 max. Sampling frame is all community heart failure nurses associated with each site
3 months post patient registration into trial	2 weeks post discharge last patient	2 months post discharge last patient	6 months post implementation	2 weeks post discharge last patient	2 months post discharge last patient	2 months post discharge last patient

* Within each site, data collection will be sequential. Across the six sites, data collection will be non-sequential as it will depend on recruitment rates at each site.

Analysis

The qualitative analysis will be undertaken using a two-step process:

Step 1 Framework analysis

The process of interpreting the transcripts will take place whilst interviews are still being conducted. This will give the research team the opportunity to explore emerging themes in detail in subsequent

For peer review only

interviews. The interviews and unstructured observations will be analysed using the Framework approach, which involves detailed familiarisation with the data, identifying key themes, interpreting the findings within the context of similar research studies, and considering policy and practice.[26] The emerging analysis will be thematic and iterative with regular discussions taking place with the process evaluation team. This involvement will support our interpretation of the interview data. The analysis will be theoretically informed by COM-B[22] and AMO.[20-22]

Step 2 Consolidated Framework for Implementation Research (CFIR)

Following initial theme generation, we will review the data using the Consolidated Framework for Implementation Research (CFIR).[18] The analysis will involve mapping barriers and facilitators onto domains within CFIR.

Quantitative data will include survey, structured observations and data on adherence to the intervention. The survey data will be entered into secure databases. Descriptive statistics will be employed to analyse survey data. Data relating to adherence to MaTI is being collected from all intervention sites. This consists of checklists detailing which components of the intervention were implemented for each patient. We will use these data to inform and explain the findings in the process evaluation sites.

Additional data

Where appropriate, we may consider using the additional data (collected for all sites as part of the wider trial) to inform, explain and triangulate findings with the process evaluation. For example, we may decide that we need more structured information on completion of the MaTI checklist to consider alongside our staff interviews and so clarify which steps of the MaTI different staff members carried out.

Additional data sources include:

- Site Feasibility Questionnaire (initial questions sent to sites to assess their suitability to take part in the trial covering areas such as clinical pathways, staffing levels/ types of specialist staff, communication with community pharmacy and the number of patients)

- Patient Experience Survey (completed by patients at two weeks/ six weeks/ twelve months post discharge)

- MaTI checklist completed in the hospital (monitors adherence to the main components of the intervention)

For peer review only

• National Heart Failure Audit Data[27] (reports on the characteristics of patients admitted with acute or sub-acute heart failure, in-hospital investigations and care, treatment given and the discharge planning and follow-up)

• Community pharmacy data collection form (describes implementation within community pharmacy)

Analysis will be integrative in order to clarify and explain the predominant systems and their implications; qualitative and quantitative data will be consolidated through a process of 'parallel mixed analysis'.[28] This includes an independent analysis of the quantitative and qualitative data to provide an understanding of key phenomena and the two understandings will be integrated using meta-inferences. For example, key findings will be generated iteratively, explicitly supported by quantitative data (such as structured observational and survey data) and substantiated or augmented by thematic qualitative data (such as interview data and field notes), and vice-versa. Survey data will be compared across the six clusters to identify any differences in staff perceptions of the barriers and facilitators to delivery. Analysis will be descriptive.

The combined analysis will therefore meet our key aims to inform interpretation of the trial findings, inform implementation of the intervention on a wider scale (for example, other long-term conditions) and aid potential future implementation of the intervention. This will be achieved by providing an in depth understanding of the overall implementation, mechanisms of impact and external factors (infra structure) that may influence delivery and functioning of the intervention.

Patient and public involvement

The ISCOMAT study has a patient-led steering group that is involved in all stages of the research process, including the process evaluation. Due to the process evaluation's iterative design we will regularly consult with the group via meetings and phone/email. The group will continue to be consulted on the research design, questions, outcome measures and findings. In particular the members of the group contribute to reviews and evaluations, as well as reading and considering study and consultation documents from a patient perspective. The group's expertise through their experiences of living with heart failure will be crucial in understanding patients' experiences with the MaTI intervention in particular.

ETHICS AND DISSEMINATION

The process evaluation has been approved as part of the ISCOMAT trial by the Research Ethics Committee and the UK Health Research Authority REC: 18/YH/0017 / IRAS: 231431.

For peer review only

Findings will be disseminated via academic and policy conferences, peer reviewed publications, social media e.g. Twitter, with further avenues for dissemination to be agreed upon with our patient led steering group.

CONCLUSION

In this paper we have described the design and methods for the mixed-methods process evaluation of the NIHR funded ISCOMAT cluster randomised controlled trial which will test the effectiveness of a complex behavioural intervention aimed at improving medications management at the interface between hospital and community for patients with hospitalised with heart failure. This process evaluation protocol demonstrates the importance of process evaluations for understanding outcomes in the clinical trial, as well as providing guidance for future process evaluations. We have followed the Medical Research Council (MRC) recommendations and guidance on the delivery of process evaluation[13] in order to support the standardisation of process evaluations.

Acknowledgements

We would like to thank Alison Blenkinsopp, Gerry Armitage, Lauren Moureau, Jan Speechley, the ISCOMAT Patient-led Steering Group, the ISCOMAT Trial Management Group, Trial Steering Committee and the Programme Steering Committee.

Competing interests statement

The authors are not aware of any competing interests.

Funding

This study is funded by the National Institute for Health Research (NIHR) [Programme Grants for Applied Research (Grant Reference Number RP-PG-0514-20009)].

Disclaimer

The views expressed are those of the authors and not necessarily those of the NIHR or the Department of Health and Social Care.

Authors' contributions

CP, LB, BF, HI, SA, CPG, PG, AF and DPA developed the detail of the process evaluation protocol. CP drafted the manuscript and all authors reviewed it critically for intellectual content and approved the final version submitted for publication.

Our collaborators include members of the wider ISCOMAT Programme Management Team who contributed to previous work packages and the ongoing programme including: Jon Silcock, David K.

For peer review only

Raynor, Robert Turner, John Wright, Ian Kellar, Roberta Longo, Ivana Holloway, Chris Bojke, Leeds Clinical Trials Research Unit.

REFERENCES

1. Dewan P, Rørth R, Jhund PS, et al. Income inequality and outcomes in heart failure: a global between-country analysis. *JACC: Heart Failure*. 2019;7(4):336-46.doi:10.1016/j.jchf.2018.11.005
2. British Heart Foundation. UK Factsheet 2020 [Available from: https://www.bhf.org.uk/what-we-do/our-research/heart-statistics. Date accessed 14/04/2020
3. Conrad N, Judge A, Tran J, et al. Temporal trends and patterns in heart failure incidence: a population-based study of 4 million individuals. *The Lancet*. 2018;391(10120):572-80.doi:10.1016/S0140-6736(17)32520-5
4. Gho JMIH, Schmidt AF, Pasea L, et al. An electronic health records cohort study on heart failure following myocardial infarction in England: incidence and predictors. *BMJ open*. 2018;8(3):e018331.doi: 10.1136/bmjopen-2017-018331
5. National Institute for Health and Social Care Excellence. Chronic heart failure in adults: diagnosis and management NICE guideline [NG106] 2018 [Available from: https://www.nice.org.uk/guidance/ng106. Date accessed 14/04/2020
6. Phipps DL, Morris RL, Blakeman T, et al. What is involved in medicines management across care boundaries? A qualitative study of healthcare practitioners' experiences in the case of acute kidney injury. *BMJ open*. 2017;7(1):e011765.doi: 10.1136/bmjopen-2016-011765
7. Urban R, Paloumpi E, Rana N, et al. Communicating medication changes to community pharmacy post-discharge: the good, the bad, and the improvements. *Int J Clin Phar,*. 2013;35(5):813-20.doi: 10.1007/s11096-013-9813-9
8. Fylan B, Marques I, Ismail H, et al. Gaps, traps, bridges and props: a mixed-methods study of resilience in the medicines management system for patients with heart failure at hospital discharge. *BMJ Open*. 2019;9(2):e023440.10.1136/bmjopen-2018-023440
9. Inamdar AA, Inamdar AC. Heart failure: diagnosis, management and utilization. *Journal of clinical medicine*. 2016;5(7):62.doi: 10.3390/jcm5070062
10. Van Spall HGC, Lee SF, Xie F, et al. Effect of patient-centered transitional care services on clinical outcomes in patients hospitalized for heart failure: the PACT-HF randomized clinical trial. *Jama*. 2019;321(8):753-61.doi: 10.1001/jama.2019.0710
11. Raynor DK, Ismail H, Blenkinsopp A, et al. Experience-based co-design—Adapting the method for a researcher-initiated study in a multi-site setting. *Health Expectations*. 2020.doi:https://doi.org/10.1111/hex.13028
12. Ellard DR, Taylor SJC, Parsons S, et al. The OPERA trial: a protocol for the process evaluation of a randomised trial of an exercise intervention for older people in residential and nursing accommodation. *Trials*. 2011;12(1):28.doi: 10.1186/1745-6215-12-28
13. Moore GF, Audrey S, Barker M, et al. Process evaluation of complex interventions: Medical Research Council guidance. *British Medical Journal*. 2015;350:h1258.doi:https://doi.org/10.1136/bmj.h1258
14. Mann C, Shaw A, Guthrie B, et al. Protocol for a process evaluation of a cluster randomised controlled trial to improve management of multimorbidity in general practice: the 3D study. *BMJ open*. 2016;6(5):e011260.doi:http://dx.doi.org/10.1136/bmjopen-2016-011260
15. Grant A, Treweek S, Dreischulte T, et al. Process evaluations for cluster-randomised trials of complex interventions: a proposed framework for design and reporting. *Trials*. 2013;14(1):15.doi:https://doi.org/10.1186/1745-6215-14-15
16. Ellard DR, Thorogood M, Underwood M, et al. Whole home exercise intervention for depression in older care home residents (the OPERA study): a process evaluation. *BMC Med*. 2014;12(1):1

For peer review only

17. Sheard L, O'Hara J, Armitage G, et al. Evaluating the PRASE patient safety intervention-a multi-centre, cluster trial with a qualitative process evaluation: study protocol for a randomised controlled trial. *Trials*. 2014;15(1):2282. doi:10.1186/1745-6215-15-420

18. Damschroder L, Aron D, Keith R. SR K, Alexander J, Lowery J. Fostering implementation of health services research findings into practice: a consolidated framework for advancing implementation science. *Implement Sci*. 2009;4:50

19. Keith RE, Crosson JC, O'Malley AS, et al. Using the Consolidated Framework for Implementation Research (CFIR) to produce actionable findings: a rapid-cycle evaluation approach to improving implementation. *Implementation Science*. 2017;12(1):15.doi:10.1186/s13012-017-0550-7

20. Macky K, Boxall P. The relationship between 'high-performance work practices' and employee attitudes: an investigation of additive and interaction effects. *The International Journal of Human Resource Management*. 2007;18(4):537-67.doi:10.1080/09585190601178745

21. Purcell J. Understanding the people and performance link: Unlocking the black box: CIPD Publishing; 2003.

22. Michie S, Van Stralen MM, West R. The behaviour change wheel: a new method for characterising and designing behaviour change interventions. *Implementation science*. 2011;6(1):42

23. Coleman EA, Mahoney E, Parry C. Assessing the quality of preparation for posthospital care from the patient's perspective: the care transitions measure. *Medical care*. 2005:246-55.doi:10.1097/00005650-200503000-00007

24. Roethlisberger F, Dickson W. Management and the Worker Cambridge, MA: Harvard University Press; 1939.

25. Campbell JP, Maxey VA, Watson WA. Hawthorne effect: implications for prehospital research. *Annals of emergency medicine*. 1995;26(5):590-4

26. Ritchie J, Lewis J, Nicholls CM, et al. Qualitative research practice: A guide for social science students and researchers: Sage; 2013.

27. National Institute for Cardiovascular Outcomes Research (NICOR). National Heart Failure Audit 2017/18 2017/18 [Available from: https://www.nicor.org.uk/national-cardiac-audit-programme/heart-failure-heart-failure-audit/. Date accessed 25/11/2019

28. Creswell JW, Clark VLP. Designing and conducting mixed methods research: Sage publications; 2017.

For peer review only

Medical Research Council process evaluation checklist[1]

Checklist	Response	Where in protocol?
**Working with policy and practice stakeholders**		
1. Are there any potential conflicts of interest arising from the relationship between evaluators and policy/practice stakeholders?	No	Page 12
2. Have the authors described how they will address these and ensure that the evaluation remains independent?	N/A	N/A
3. Does the proposal set out a clear plan for communicating findings to policy and practice stakeholders during the evaluation?	Yes	Ethics and dissemination on page 11
**Relationships between evaluation components**		
4. Is the relationship between the process evaluation and other evaluation components clearly defined and justified?	To an extent. More detail to come in results paper	'The process evaluation' on page 4
5. Will process and outcomes evaluation be conducted by the same team or by separate teams?	Separate teams	'The process evaluation' on page 4
6. If the former, how will researchers ensure that knowledge of outcomes or process does not bias analysis of the other?	N/A	N/A
7. If the latter, is there clear oversight of the two components?	Yes	'Process evaluation' on page 4 (regular meetings)
8. Is it clear that the principal investigator values all aspects of the evaluation, and will provide effective oversight of all aspects of the evaluation?	Yes	'Process evaluation' on page 4 (regular meetings)
**Intervention description and theory**		
9. Is the intended intervention fully described?	No	'Study design' on page 5 indicates that we can only provide limited information
10. Are standardised terminology and definitions of intervention components adopted where possible?	As above	As above
11. Are the structures and processes involved in intervention delivery fully described?	As above	As above
12. If appropriate, will a full intervention manual be made publicly available?	Yes if intervention is successful	N/A for protocol paper
13. Is a clear, plausible, set of causal assumptions specified and justified (for example, in a logic model)?	To be described in a separate paper	N/A
14. Does this draw upon appropriate	Yes	Page 5 'Study design' AMO, COM-B

For peer review only

theories?		and CFIR
15. If not, are there plans to develop a theory as part of the research?	N/A	N/A
16. Have the authors planned to review these assumptions with policy and practice stakeholders to explore agreement and divergence on what the intervention is, and how it will work?	Yes	Completed and discussed in another paper
**Process evaluation aims and research questions**		
17. Are the research questions clear, important and well justified with reference to the theory of the intervention and the status of the evidence base? What decisions will they inform?	Yes	Research questions and objectives on page 4. Justified on page 3 in 'Introduction'.
18. Have the authors considered whether previous process evaluations have been conducted of interventions involving similar components or theories of change?	Yes	Literature highlighted in 'Study design' on page 5
19. Have they adopted comparable aims and methods, or justified not doing so?	Yes	Methods outlined from page 5
20. Has the theory of the intervention (or logic model) been used to identify key areas of uncertainty for investigation by process evaluation?	Yes	Yes CFIR AMO and COM-B will be included in data collection and analysis processes as highlighted on page 5
21. Have the authors considered which components may prove most challenging to implement (e.g. which represent more fundamental change, or for which there is least agreement on what they are and the purposes they serve)?	Yes	Detail to be provided in results paper
22. Have the authors considered for which causal assumptions evidence is most equivocal?	Yes	To be reported in results paper
23. How will unanticipated consequences be captured?	Yes	To be reported in results paper. Changes from protocol will be recorded in a table
24. Is there linkage between research aims? Do they fit together to address the overall study aim?	Yes	Study aims to be found on page 4
25. If conducted alongside an outcomes evaluation, is the added value of the process evaluation explained? Is it clear how the research will enhance the interpretation of outcomes?	Yes	Explained in 'Study design' page 5
26. Will process evaluation provide sufficient assurances regarding the internal validity of the outcomes	Yes	Examining data from the trial, e.g. Hospital (MaTI) checklist (monitors adherence to the main components of

For peer review only

evaluation?		the intervention) Page 10
27. Will it enable policymakers/practitioners to understand how the intervention might be applied in different contexts?	N/A protocol paper	To be reported in results paper
28. Have the authors stated how and when they will combine process and outcomes data?	N/A protocol paper	To be reported in results paper
**Selection of methods to address research questions**		
29. Are the quantitative and qualitative methods selected appropriate to the research questions?	Yes	Pages 5 to 11 discuss methods used. More detail will be provided in results paper
30. Will implementation be captured in sufficient detail to establish consistency with the theory of the intervention?	N/A protocol paper	To be reported in results paper
31. Are existing validated measures used where possible? Are plans to validate new measures included?	Yes	Page 9 on framework analysis and CFIR
32. How will emerging changes, adaptations or additions to the intervention be captured?	N/A protocol paper	N/A
33. Are the quantitative methods appropriate? (e.g. 'tick box' self-report by implementers of intervention delivery should be avoided if possible).	Yes	Page 6-11 we describe quantitative methods
34. Are the qualitative methods appropriate?	Yes	Pages 6-11 we describe qualitative methods
35. Have the authors considered how change in practice as a result of being observed or measured will be minimised?	Yes	Page 6 on hawthorn effect
36. Have the authors considered the timing of data collection, and its impact on the data collected?	Yes	Page 8-9 on timing of data collection
37. Have the authors investigated whether any routine programme monitoring data can be used? If so, are there plans to check their validity and reliability?	Yes	Interviews will explore routine data such as MaTI checklists to explain if there are poor quality results. Page 11
38. Have the authors stated how quantitative and qualitative methods will be combined?	Yes	Page 10-11 Greater detail will be given in results paper
39. Have the authors considered how they will respond if challenges emerge during the evaluation - for example, if serious implementation failures are identified which need deeper investigation?	Yes	We describe possible challenges carrying out observations and how this will be addressed. Page 6
**Resource considerations in collecting/analysing process data**		
40. Who will lead or conduct the process evaluation? Do they have,	Yes	The lead is named on the paper

For peer review only

or have direct access to, appropriate expertise and experience?		
41. Does the research team have sufficient expertise in quantitative and qualitative methods and relevant social science theory?	Yes	N/A
42. Is sufficient time, funding and staff resource included for data collection, analysis (including sufficient time to conduct good quality analysis of qualitative data, with quality checks by a second coder where appropriate) and reporting?	Yes	Considered regularly at meetings
**Analysis and reporting**		
43. Has consideration been given to the use of quantitative process measures for modelling variations in outcomes and/or cost-effectiveness?	Yes	Separate cost evaluation is being conducted
44. Is the relationship between qualitative data components and outcomes and/or cost effectiveness analysis clear?	Yes	Relationship between qualitative data and outcomes discussed on page 11. Separate cost evaluation is being conducted.
45. Is there a coherent strategy for dissemination to an academic audience and wider stakeholders?	Yes	Page 11. Discussed at regular meeting.

1. Moore GF, Audrey S, Barker M, et al. Process evaluation of complex interventions: Medical Research Council guidance. *British Medical Journal*. 2015;350:h1258.doi:https://doi.org/10.1136/bmj.h1258

For peer review only
